# Microsatellite resources of *Eucalyptus*: current status and future perspectives

**DOI:** 10.1186/s40529-014-0073-3

**Published:** 2014-10-25

**Authors:** Murugan Sumathi, Ramasamy Yasodha

**Affiliations:** Division of Plant Biotechnology, Institute of Forest Genetics and Tree Breeding, Coimbatore, 641 002 India

**Keywords:** Eucalyptus, Microsatellites, EST-SSRs, miRNA-SSRs, Genotyping, Population genetics, Marker assisted selection

## Abstract

*Eucalyptus* is the premier paper pulp, short rotation plantation species grown all over the world. Genetic improvement programs integrating molecular marker tools are in progress in many parts of the globe to increase the productivity. Whole genome sequence and expressed sequence tags (ESTs) of the eucalypts paved way for introduction of molecular genetics and breeding in this genus. Different molecular characterization approaches have been used simultaneously in eucalypts, however, microsatellites or simple sequence repeats (SSRs) with their prolific characteristics could occupy a special niche in *Eucalyptus* genetic improvement. Further, highly informative SSRs were used for the clonal identity, genetic fidelity and in certification of breeder’s rights. *Eucalyptus* genetic linkage maps generated with microsatellite loci were used successfully to identify quantitative trait loci (QTLs) for various economically important traits. Progressively more numbers of microsatellites are being linked to genes associated with adaptive and functional variations, therefore making their utility broader in genetic applications. Availability of common SSR markers across the species provides an opportunity to validate the expression of QTLs across variable genetic backgrounds and accurately compare the position of QTLs in other species. Recent evidences suggest that the presence of SSRs in micro RNAs of plant species play a role in the quantitative trait expression. Similar studies in eucalypts may provide new insights into the genetic architecture of transcript-level variations and post transcriptional gene regulation. This review on eucalypts microsatellites, highlights the availability and characteristics of genomic and eSSRs and their potential in genetic analysis of natural and breeding populations and also discusses the future prospects in population genetics and marker assisted selection.

**Electronic supplementary material:**

The online version of this article (doi:10.1186/s40529-014-0073-3) contains supplementary material, which is available to authorized users.

## Review

### Introduction

*Eucalyptus* is the world’s leading industrial plantation species due to its fast growth, wider adaptability and multipurpose utility. Until lately, most of the *Eucalyptus* plantations all over the world were destined for paper production, however, presently these plantations are looking forth to support solid wood industry including veneer production (Luo et al. [2013]). Research and development inputs on eucalypts have quadrupled the plantation productivity with the current norm of 40 m^3^/ha/yr (ABRAF [2013]), and unleashed possibilities up to 100 m^3^/ha/yr with intensive management (Evans and Turnbull [2004]). Efficient breeding and clonal deployment strategies have been employed for improved planting stock development. Efforts on molecular breeding and molecular genetic analysis are underway in eucalypts to accelerate breeding and conservation. Different kinds of DNA markers have been employed for a variety of purposes, including population genetics and marker assisted selection (MAS). One of the most frequently used DNA markers in eucalypts since 1996 are microsatellites or simple sequence repeats (SSRs) (Byrne et al. [1996]). SSRs possess characteristics such as ubiquitous distribution in genome, locus specificity, co-dominance, multi allelism, high mutation rate, heterozygous, transferability across species and associated with the gene expression and function. Hence, these markers are considered to be ideal for conservation genetics, genetic diversity assessment, variety protection, and construction of high-resolution genetic maps to link phenotypic and genotypic variation. The importance of microsatellites for plant genome analysis has been highlighted on several occasions (Varshney et al. [2005]; Kalia et al. [2011]). Earlier, genomic SSRs (gSSRs) were developed by isolating and sequencing clones containing putative SSR regions, which are costly and time consuming. Subsequently, development of online databases like GenBank led to the generation of Expressed Sequence Tag (EST) derived-SSRs (eSSRs) which are present in transcribed regions of the genome. However, in recent times, tremendous data on gSSRs and eSSRs are made available through next generation sequencing (NGS) methods (Zalapa et al. [2012]; Kudapa et al. [2014]) which can readily be used in population genetics and breeding applications.

In eucalypts, primarily genomic SSRs were developed for very few commercially important species like *E. urophylla, E. grandis, E. globulus* and *E. nitens.* However, high synteny of genome existing across eucalypt species has benefited many DNA data deficient eucalypts taxa, thus excellent SSR transferability was witnessed across species (da Silva et al. [2009]; Acuna et al. [2012a]). SSRs were used in eucalypts for multiple purposes such as species identification, phylogeny, hybrid authenticity, genetic diversity studies, genetic mapping and Quantitative trait loci (QTL) localization (Myburg et al. [2007]; Grattapaglia et al. [2012]). SSRs also offer a much higher level of automation which is crucial to manage with the great number of individuals generally handled in eucalypt breeding. These markers have advantages over high throughput SNP markers, because the majority of SNPs is bi-allelic and information (heterozygosity) generated is low, whereas SSR mutational rates are a lot higher and the DNA slippage process creates a number of new alleles leading to the generation of maximal information (Ellegren [2004]). Many studies on the comparison between SSRs and SNPs have proven that large numbers of SNP loci were required to replace highly polymorphic SSRs in studies of diversity and relatedness (Hamblin et al. [2007]; Yang et al. [2011]). Further, SSR markers were always preferred as framework markers for developing consensus linkage map, composite integrated linkage map and comparative map between the species (Garcia et al. [2011]; Hudson et al. [2012]).

### Microsatellite resources in eucalypts

Based on the published literature, till date, a total of 505 genomic SSRs (gSSRs) and 758 validated EST-SSRs (eSSRs), 35 chloroplast SSRs (cpSSRS) and 8 gene based SSRs (CG-SS1Rs) have been applied in different species of *Eucalyptus*. The details on SSR marker code, source species and number of SSRs developed are given in the Table 1. A largest collection of both gSSRs and eSSRs (~300 SSRs) was developed from *E. grandis* and *E. urophylla* with the prefix as Embra (Brondani et al. [1998], [2002], [2006]; Faria et al. [2010], [2011]). Other major resources include the SSRs with the prefix Emcrc (40 SSRs) developed from *E. globulus* (12 loci; Steane et al. [2001]), *Corymbia variegata* (14 loci; Jones et al. [2001]) and *Corymbia citriodora* subsp *variegata* (14 loci; Shepherd et al. [2006]). The SSRs with prefixes En, Es, Eg and El were developed from species such as *E. nitens* (8 SSRs), *E. sieberi* (8 SSRs), *E. globulus* (26 SSRs) and *E. leucoxylon* (13 SSRs) respectively (Byrne et al. [1996]; Glaubitz et al. [2001]; Ottewell et al. [2005]). A set of 35 chloroplast DNA microsatellites was developed based on the full cp-DNA sequence of *E. globulus* (Steane et al. [2005]). The ISSR-enrichment technique was exploited for the development of five SSR loci in *E. grandis* (Van der Nest et al. [2000]). Very few SSR loci were developed from *E. urophylla* and *E. pilularis* (Payn et al. [2008]; Sexton et al. [2010]). NGS methods such as 454 sequencing was applied to isolate ten SSRs from *E. victrix* (Nevill et al. [2013]). A recent study in *Eucalyptus* found that microsatellites occupy approximately 0.6 percent of the overall genome (Ranade et al. [2014]).

**Table 1:** **List of genomic and chloroplast SSRs developed in** ***Eucalyptus***

S.NO	SSR code	Name of the species	Number of SSRs developed	Reference
1	En	*E. nitens*	8	Byrne et al. [1996]
2	EMBRA	*E. grandis* × *E. urophylla*	300	Brondani et al. [1998], [2002], [2006]
3	FMRSA	*E. grandis*	5	Van der Nest et al. [2000]
4	EMCRC	*C.variegata*	14	Jones et al. [2001]
5	EMCRC	*E. globulus*	12	Steane et al. [2001]
6	Es	*E. sieberi*	8	Glaubitz et al. [2001]
7	Eg	*E. globulus*	26	Thamarus et al. [2002]
8	El	*E. leucoxylon*	13	Ottewell et al. [2005]
9	EMCRC cp	*E. globulus*	35	Steane et al. [2005]
10	EMCRC	*C. citriodora subsp variegata*	14	Shepherd et al. [2006]
11	FMG EUC SSR	*E. urophylla*	3	Payn et al. [2008]
12	EC	*E. camaldulensis*	14	da silva et al. [2009]
13	EPIL	*E. pilularis*	2	Sexton et al. [2010]
14	KPEV	*E. victrix*	10	Nevill et al. [2013]
15	EMBRA	*E. grandis*	41	Grattapaglia et al. [2014]

Whole-genome sequencing of *E. camaldulensis* and *E. grandis* have been completed by the Kazusa DNA Research Institute, Japan and DOE Joint Genome Institute (JGI), USA in collaboration with members of the *Eucalyptus* Genome Network (EUCAGEN) respectively (Hirakawa et al. [2011]; Myburg et al. [2014]). Additionally, transcriptome resources were generated from various tissues including xylem, phloem, root, shoot, leaf and reproductive tissues from species such as *E. grandis*, *E. gunnii*, *E. globulus*, *E. camaldulensis* and *E. tereticornis* (Mizrachi et al. [2010]; http://web.up.ac.za/eucagen/; http://eucgenie.org/; Healey et al. [2014]). All these transcriptome resources have promoted the development of SSR markers *in silico* and many of which were used for diverse purposes across eucalypt species (Ceresini et al. [2005]; Rabello et al. [2005]; Yasodha et al. [2008]; Rengel et al. [2009]; He et al. [2012]; Zhou et al. [2014]). Recently, gene specific microsatellites were developed from *E. grandis, E. globulus* and *E. gomphocephala* (Acuna et al. [2012b]; Bradbury et al. [2013a]). Table 2 provides the list of EST-SSRs developed in various species of eucalypts.

**Table 2:** **List of EST- SSRs developed in** ***Eucalyptus***

S.NO	SSR code	Name of the species	Number of SSRs developed	Reference
1	EST - SSR	*E. globulus*	3	Yasodha et al. [2008]
2	EST - EMBRA	*E. grandis*	20	Faria et al. [2010]
3	EST - EMBRA	*E. grandis*	21	Faria et al. [2011]
4	EUCeSSR Candidate EST-SSR	*E. gunnii*	32	Zhou et al. [2010]
5	EST	*E. globulus*	37	Acuna et al. [2011]
6	SSR - CG	*E. globulus*	8	Acuna et al. [2012b]
7	EUCeSSR	*E. globulus, E. gunnii, E. grandis, E. tereticornis, E. grandis* × *E. nitens*	198	He et al. [2012]
8	EUCeSSR	*E. grandis*	240	Zhou et al. [2014]
9	EGM	*E. gomphocephala*	17	Bradbury et al. [2013a]
10	EST - EMBRA	Multi-species collection	453	Grattapaglia et al. [2014]

The frequency of occurrence of microsatellites had varied in different databases of *Eucalyptus* for example, 12.9% in NCBI database (Yasodha et al. [2008]) 13.3% in EUCAWOOD (Rengel et al. [2009]), 25.5% and 29% in FORESTs database (Rabello et al. [2005]; Ceresini et al. [2005]). The type of SSRs found in the ESTs varied among the transcriptome analyzed. In general, amongst the SSR motifs, the dimeric and trimeric were most abundant followed by other types. This is in consonance with many other plant species belonging to monocots as well as dicots. The most represented di-nucleotide was AG/TC (72.5%) motif followed by the trimeric CCG/GGC, AAG/TTC, and AGA/TCT (12.81%) (Ceresini et al. [2005]; Rabello et al. [2005]; Yasodha et al. [2008]; Rengel et al. [2009]; Ranade et al. [2014]). The details on different types of motifs are shown in Table 3. These motifs have also been found to be predominant dinucleotide repeats and trinucleotide repeats respectively in many plant species (Zhou et al. [2014]). The BAC clones of eucalypts had comparatively less numbers of SSR frequency (Paiva et al. [2011]). Most of the SSRs isolated from genomic libraries were targeted for simple dinucleotide repeats, particularly AG/TC motifs except a few had compound, interrupted and trinucleotide motifs (Glaubitz et al. [2001]; Ottewell et al. [2005]; Brondani et al. [2006]). Unique *in silico* methods were developed to extract and transfer the highly conserved orthologus genic SSR regions from *E. globulus* to *E. camaldulensis*, a species with less genomic information, and such novel SSRs were useful for parentage analysis, confirmation of interspecific hybrid and genotyping of seedling seed orchard (Nagabhushana et al. [2011]). A study to mine SSRs *in silico* from 22298 EST sequences of eucalypts revealed that primers could be designed for 1244 microsatellites, of which 182 were selected for characterization based on polymorphism status among species (Grattapaglia et al. [2014]).

**Table 3:** **Types of SSR motifs in** ***Eucalyptus*** **species**

S.NO	Types of SSR motifs	Percentage	Major nucleotide motifs		Reference
			Motif	Percentage	
1	DNRs	37.0	AG/CT	35.2	Ceresini et al. [2005]
	TNRs	33.0	CCG/GGC	12.8	
2	DNRs	41.0	AG/CT	94.4	Rabello et al. [2005]
	TNRs	36.1	CCG/GGC	37.9	
3	DNRs	50.9	AG/TC	90.0	Yasodha et al. [2008]
	TNRs	45.0	GGC/CCG	17.0	
4	DNRs	29.4	AG/TC	87.8	Rengel et al. [2009]
	TNRs	46.3	AAG/TTC	32.3	
			AGA/TCT		
			GAA/CTT		

### Cross-species transferability

Microsatellite markers are generally transferable across related genera and the genetic distance among the species limits the percent transferability. The cross species transferability provides a potential source of codominant markers for many related species and facilitates evolutionary, ecological, and conservation studies across the species. Since the display of cross-species transferability of microsatellite markers is high in eucalypts, attempts have been made to identify a useful number of primer sets of high utility in a wide range of species. Details of the SSR source species and the species in which the SSRs cross amplified are given in the Table 4. The cross species transferability of genomic SSR markers was comparatively higher among the species of *Eucalyptus* and the transferability rate dropped down in the species of *Corymbia* and *Angophora* (Steane et al. [2001]). Initially, Byrne et al. ([1996]) tested transferability of four nuclear microsatellite markers from *E. nitens* and found that they were transferable (50%) to sub genera *Symphyomyrtus* and *Monocalyptus* but not to the genus *Corymbia*. However, modifications in the PCR amplification protocols could improve the transfer rate across genera (Shepherd et al. [2006]). Many of the SSR loci isolated from *E. grandis* and *E. urophylla* were cross amplified in different species like *E. globulus*, *E. nitens*, *E. pilularis*, *E. urophylla*, *E. pyrocarpa*, *E. camaldulensis* and *E. tereticornis* (Bundock et al. [2000]; Glaubitz et al. [2001]; Steane et al. [2001]; Agrama et al. [2002]; Ottewell et al. [2005]; Arumugasundaram et al. [2011]; Subashini et al. [2013]). Similarly, the eSSRs were successfully cross amplified in several species like *E. grandis*, *E. saligna*, *E. dunnii*, *E. viminalis*, *E. camaldulensis*, *E. urophylla* and *E. tereticornis* (Neves et al. [2011]; Faria et al. [2010], [2011]; Acuna et al. [2012b]; Hudson et al. [2012]; Petroli et al. [2012]; Breed et al. [2012]; He et al. [2012]; Bradbury et al. [2013a], [b]; Bradbury and Krauss [2013]). Recently, gene-homologous eSSRs designed for *E. gomphocephala* were transferred to *E. marginata*, *E. camaldulensis*, and *E. victrix* (Bradbury et al. [2013a]).

**Table 4:** **Details on the** ***Eucalyptus*** **species used for SSR development and species showed cross transferability**

S.No	Species used for SSR development	Species transferred	Reference
1	*E. globulus*	*E. grandis*	Neves et al. [2011]; Alves et al. [2011]; Kullan et al. [2011], [2012]; Acuna et al. [2012a]; Hudson et al. [2012]; Bartholome et al. [2013]
		*E. saligna, E. dunnii*	Alves et al. [2011]; Acuna et al. [2012a]
		*E. viminalis*	Acuna et al. [2012a]
		*E. camaldulensis*	Butcher et al. [2009]; Alves et al. [2011]; Acuna et al. [2012a]; Bradbury et al. [2013a]; Subashini et al. [2013]; Wheeler et al. [2013]
		*E. tereticornis*	Alves et al. [2011]; He et al. [2012]; Acuna et al. [2012a]; Subashini et al. [2013]
		*E. urophylla*	Alves et al. [2011]; Neves et al. [2011]; Kullan et al. [2011], [2012]; Hudson et al. [2012]; He et al. [2012]; Bartholome et al. [2013]
		*E. obliqua*	Nevill et al. [2008], [2010]; Bloomfield et al. [2011]
		*E. gomphocephala*	Bradbury and Krauss [2013]; Bradbury et al. [2013a]; Wheeler et al. [2013]
		*E. victrix, E. marginata*	Bradbury et al. [2013a]
		*E. benthamii*	Butcher et al. [2005]
		*E. nitens*	Steane et al. [2001]; Henery et al. [2007]; Grosser et al. [2008]; Barbour et al. [2010]; Thumma et al. [2010]
		*E. regnans, E. delegatensis, E. pauciflora, E. radiata, E. cloeziana*	Nevill et al. [2008], [2010]
		*E. bicostata, E. maidenii*	Steane et al. [2001]
		*E. perriniana* × *E. nitens*	Barbour et al. [2010]
		*E. aggregata, E. rubida,*	Field et al. [2010]
		*E. microtheca*	Mamaghani et al. [2010]
		*E. decipiens, E. rudis, E. caladocalyx*	Wheeler et al. [2013]
2	*E. grandis and E. urophylla*	*E. camaldulensis*	Agrama et al. [2002]; Arumugasundaram et al. [2011]; Alves et al. [2011]; Bradbury et al. [2013a]; Subashini et al. [2013]
		*E. obliqua*	Nevill et al. [2008]; Bloomfield et al. [2011];
		*E. gomphocephala*	Bradbury and Krauss [2013]; Bradbury et al. [2013a]
		*E. victrix, E. marginata*	Bradbury et al. [2013a]
		*E. globulus*	Bundock et al. [2000]; Glaubitz et al. [2001]; Marques et al. [2002]; Thamarus et al. [2002]; Patterson et al. [2004]; Freeman et al. [2006]; Foster et al. [2007]; Jones et al. [2007]; Nevill et al. [2008]; Freeman et al. [2008a], [b]; Rao et al. [2008]; Freeman et al. [2009]; Alves et al. [2011]; Ribeiro et al. [2011]; Hudson et al. [2012]; Freeman et al. [2013]; Jones et al. [2013]
		*E. benthamii*	Butcher et al. [2005]
		*E. grandis*	Chaix et al. [2003]; Missiaggia et al. [2005]; Kirst et al. [2005b]; Jones et al. [2008]; Rosado et al. [2010]; Alves et al. [2011]; Gion et al. [2011]; Kullan et al. [2011]; Neves et al. [2011]; Kullan et al. [2012]; Hudson et al. [2012]; Petroli et al. [2012]; Bartholome et al. [2013]
		*E. consideniana, E. sieberi*	Glaubitz et al. [2001], [2003]
		*E. nitens*	Glaubitz et al. [2001]; Grosser et al. [2008]
		*E. brownii, E. populnea*	Holman et al. [2003]
		*E. urophylla*	Tripiana et al. [2007]; Payn et al. [2008]; Rosado et al. [2010]; Gion et al. [2011]; Kullan et al. [2011]; Neves et al. [2011]; Alves et al. [2011]; Kullan et al. [2012]; Hudson et al. [2012]; Petroli et al. [2012]; Bartholome et al. [2013]
		*E. regnans, E. radiata, E. delegatensis, E. pauciflora*	Nevill et al. [2008], [2010]
		*E. cloeziana*	Stokoe et al. [2001]; Nevill et al. [2008]
		*E. loxophleba*	Sampson and Byrne [2008]
		*E. tereticornis*	Marques et al. [2002]; Alves et al. [2011]; Arumugasundaram et al. [2011]; Subashini et al. [2013]
		*E. curtissii*	Smith et al. [2003]
		*E. dunnii*	Poltri et al. [2003]; Zelener et al. [2005]; Alves et al. [2011]
		*E. microtheca*	Mamaghani et al. [2010]
		*E. perriniana*	Rathbone et al. [2007]
		*E. pilularis*	Shepherd et al. [2010]
		*C. torelliana* × *C. citriodora subsp variegata*	Shepherd et al. [2006], [2008]
		*E. acmenoides* × *E. cloeziana*	Stokoe et al. [2001]
		*E. aggregata* × *E. rubida*	Field et al. [2010]
3	*E. nitens*	*E. urophylla*	Neves et al. [2011]; Kullan et al. [2011]; He et al. [2012]; Hudson et al. [2012]; Kullan et al. [2012]; Bartholome et al. [2013]
		*E. grandis*	Byrne et al. [1996]; Kullan et al. [2011]; Neves et al. [2011]; Kullan et al. [2012]; Hudson et al. [2012]; Bartholome et al. [2013]
		*E. benthamii*	Butcher et al. [2005]
		*E. camaldulensis*	Byrne et al. [1996]; Butcher et al. [2009]
		*E. globulus*	Byrne et al. [1996]; Bundock et al. [2000]; Thamarus et al. [2002]; Freeman et al. [2006], [2008b]; Hudson et al. [2012]; Freeman et al. [2013];
		*E. sieberi, E. marginata, E. maculata*	Byrne et al. [1996]
		*E. tereticornis*	He et al. [2012]
4	*E. leucoxylon*	*E. obliqua*	Bloomfield et al. [2011]
		*E. gomphocephala, E. camaldulensis, E. victrix, E. marginata*	Bradbury et al. [2013a]
		*E. nitens*	Ottewell et al. [2005]; Henery et al. [2007]; Thumma et al. [2010]
		*E. regnans, E. obliqua, E. delegatensis, E. pauciflora, E. radiata, E. cloeziana*	Nevill et al. [2008]
		*E. globulus, E. sieberi*	Ottewell et al. [2005]
5	*E. sieberi*	*E. obliqua*	Nevill et al. [2008]; Bloomfield et al. [2011]
		*E. benthamii, E. camaldulensis*	Butcher et al. [2005], [2009]
		*E. nitens*	Glaubitz et al. [2001]; Henery et al. [2007]; Thumma et al. [2010]
		*E. regnans, E. delegatensis, E. pauciflora, E. radiata, E. cloeziana*	Nevill et al. [2008]
		*E. globulus*	Glaubitz et al. [2001]; Thamarus et al. [2002]
		*E. urophylla, E. grandis*	Neves et al. [2011]; Kullan et al. [2011], [2012]
		*C. torelliana, C. citriodora subsp variegata*	Shepherd et al. [2008]
6	*E. gunnii*	*E. gomphocephala*	Bradbury and Krauss [2013]; Bradbury et al. [2013a]; Wheeler et al. [2013]
		*E. camaldulensis, E. victrix, E. marginata*	Bradbury et al. [2013a]; Wheeler et al. [2013]
		*E. urophylla and E. tereticornis*	He et al. [2012]
		*E. decipiens, E. rudis, E. cladocalyx*	Wheeler et al. [2013]
7	*E. grandis*	*E. incrassata*	Breed et al. [2012]
		*E. camaldulensis*	Butcher et al. [2009]; Faria et al. [2010], [2011]; Wheeler et al. [2013]
		*E. saligna, E. dunnii, E. grandis*	Faria et al. [2010], [2011]
		*C. torelliana* × *C. citriodora subsp variegata*	Shepherd et al. [2006]
		*E. tereticornis*	He et al. [2012]
		*E. gomphocephala, E. decipiens, E. rudis, E. cladocalyx*	Wheeler et al. [2013]
		*E. urophylla*	Faria et al. [2010], [2011]; He et al. [2012]
		*E. globulus*	Freeman et al. [2008a]; Faria et al. [2010], [2011];
8	*E. camaldulensis*	cross ampliefied in 25 different species	da Silva et al. [2009]
9	*Eucalyptus*	*E. tessellarii, E. curtisii, E. citriodora, E. tetrodonta, E. cloeziana, E. regnans, E. grandis*	Zhou et al. [2014]

### Genotyping of SSRs and allele variations in eucalypts

Genotyping studies with microsatellites generally preferred dinucleotide repeats because they bring off high polymorphism and display more variation among individuals. In certain occasions the dinucleotide repeats based allelic variation results in shadow bands or stutter bands during electrophoresis thus leading to genotyping errors (Hoffman and Amos [2005]). Hence, the genotyping applications requiring high precision like clonal certification, microsatellite markers with tetra and penta nucleotide motifs were recommended (Faria et al. [2010]). Loci with higher length repeats provided an advantage of accurate allele calling due to their larger allele size difference. In eucalypts, presence of tetra, penta and hexanucleotide allowed easy allele calling which was challenging when di and trinucleotide motif SSRs were used (Faria et al. [2010]). Presence of null allele, i.e., a microsatellite locus that consistently fails to amplify to detected levels via the polymerase chain reaction (PCR) is not uncommon in eucalypts (Glaubitz et al. [2001]). In general, microsatellite null alleles at low frequencies are unlikely to introduce serious biases into population genetic analysis (Dakin and Avise [2004]). Nevertheless, microsatellite null alleles can cause egregious errors when they are used for genetic mapping experiments at family level. There are methods and software packages available to handle the null alleles and heterozygote deficiency in various genetic analyses (Chapuis and Estoup [2007]; Chybicki and Burczyk [2009]). In a genetic mapping study, it was found that 20 out of 241 segregating SSR loci were observed to have null alleles (Brondani et al. [2006]). Increased occurrence of null alleles have been observed when attempting to transfer microsatellites across related species (Faria et al. [2010]; Bradbury et al. [2013a], [b]; Bradbury and Krauss [2013]) and appropriate strategies need to be used in handling such data. Genotyping with microsatellites markers on large scale using DNA sequencing instruments demonstrated very high accuracy of allele sizing and binning to avoid the scoring errors. Although various methods of primer labeling and SSR allele detection in eucalypts was reported (Ottewell et al. [2005]; Missiaggia and Grattapaglia [2006]; Faria et al. [2011]; Subashini et al. [2013]), difficulties in accuracy of allele sizing continue to exist.

Allele sizes generated by the microsatellites loci have larger implications on genotyping of the individuals. Allele sizes generated by the eucalypt microsatellite loci were highly varying, as low as 50 bp was detected in *E. camaldulensis* (da Silva et al. [2009]). Very high levels of allelic variability were observed at different levels of populations. A pioneering study on analysis of 15 SSR loci in 32 F1 individuals of *E. grandis* × *E. urophylla* generated 9 to 26 alleles per locus with an average of 16.3 × 4.8 (Brondani et al. [1998]). Similarly, a breeding population of *E. grandis* with 192 selected individuals produced a total of 119 alleles with 6 SSR loci, yielding a minimum of 6 (Embra11) and a maximum of 33 alleles (Embra16), with an average of 19.8 × 9.2 alleles per locus (Kirst et al. [2005a]).

Compared to gSSRs, eSSR loci were known for low levels of polymorphism, Faria et al. ([2010]) analyzed 10 eSSRs in 6 eucalypt species wherein the number of alleles were in the range of 7–15 (*E. grandis*), 5–12 (*E. globulus*), 4–10 (*E. urophylla*), 6–14 (*E. camaldulensis*), 5–9 (*E. dunnii*) and 4–14 (*E. saligna*). Allele size variations favored multiplexing of fluorescent based automated DNA genotyping applications, wherein upto 18 loci were analyzed in a single run with 5 dye format (Faria et al. [2010]).

**Figure 1:**
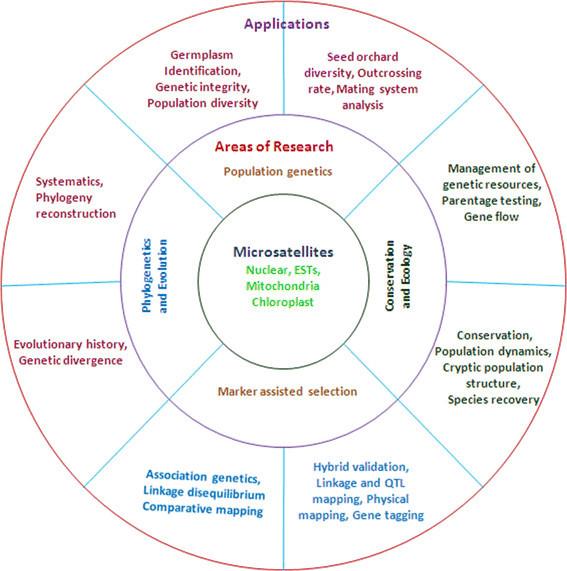
**Areas of research and applications of microsatellites in** ***Eucalyptus*** **.**

*Eucalyptus* microsatellites are usually highly informative as revealed through the statistics of polymorphic information content (PIC) and heterozygosity (He) (Table 5). The most widely used SSRs such as Embra, generated maximum heterozygosity value of 0.95 (Holman et al. [2003]; Jones et al. [2008]), Emcrc markers showed highest heterozygosity of 0.92, Es, En and El series loci produced 0.95, 0.91 and 0.93 (Byrne and Hines [2004]; Glaubitz et al. [2001]; Ottewell et al. [2005]). Accordingly the PIC values were also very high (0.933) in most of the studies so far reported (Kirst et al. [2004]). Because of these characteristics of microsatellites, they were used for various purposes in plant genome analysis (Figure 1).

**Table 5:** **Characteristics of major eucalypt SSR loci applied in population genetic studies**

S.No	Species	Na (Minimum–Maximum)	Ho (Minimum–Maximum)	He (Minimum–Maximum)	Reference
1	*E. nitens*	9–16	0.40–0.80	0.72–0.91	Byrne et al. [1996]
2	*E. grandis*	5–18	0.33–0.87	0.74–0.91	Brondani et al. [1998]
3	*E. urophylla*	7–17	0.35–0.81	0.60–0.93	Brondani et al. [1998]
4	*E. globulus*	14–21	0.31–0.85	0.69–0.92	Steane et al. [2001]
5	*E. globulus*	16–24	0.42–0.78	0.81–0.92	Jones et al. [2002]
6	*E. urophylla*	5–16	0.1–0.93	0.23–0.93	Brondani et al. [2002]
7	*E. grandis*	4–17	0.18–0.93	0.64–0.93	Brondani et al. [2002]
8	*E. curtisii*	1–9	0.0–0.85	0–0.85	Smith et al. [2003]
9	*E. brownii, E. populnea*	10–21	0.61–1	0.58–0.95	Holman et al. [2003]*
10	*E. grandis*	14–21	0.51–0.85	0.62–0.86	Chaix et al. [2003]
11	*E. dunnii*	11–22	0.26–0.83	0.68–0.93	Zelener et al. [2005]
12	*E. benthamii*	4–26	0.61–0.71	0.62–0.78	Butcher et al. [2005]
13	*E. leucoxylon*	8–20	0.77–0.92	0.47–0.93	Ottewell et al. [2005]
14	*E. grandis*	6–33	-	0.65–0.94	Kirst et al. [2005b]
15	*E. globulus*	4.6–9.6	0.66–0.74	0.66–0.79	Jones et al. [2006]*
16	*E. globulus*	9.7	0.62	0.75	Freeman et al. [2007]*
17	*E. perriniana*	5.6–10.9	0.54–0.72	0.65–0.83	Rathbone et al. [2007]*
18	*E. globulus*	9–21	0.55–0.83	0.72–0.91	Foster et al. [2007]
19	*E. grandis*	14–28	0.59–0.93	0.77–0.95	Jones et al. [2008]
20	*E. regnans*	4–15	0.65–0.91	0.67–0.93	Nevill et al. [2008]
21	*E. obliqua*	5–20	0.57–0.94	0.63–0.94	Nevill et al. [2008]
22	*E. delegatensis*	6–19	0.68–0.94	0.59–0.93	Nevill et al. [2008]
23	*E. radiata*	6–17	0.65–0.95	0.7–0.91	Nevill et al. [2008]
24	*E. pauciflora*	5–15	0.6–0.88	0.67–0.9	Nevill et al. [2008]
25	*E. cloeziana*	5–10	0.62–0.82	0.57–0.85	Nevill et al. [2008]
26	*E. globulus*	11–17	0.75–0.83	0.85–0.90	Rao et al. [2008]
27	*E. camaldulensis*	5–13	0.28–0.84	0.25–0.9	da Silva et al. [2009]
28	*E. camaldulensis*	5.0–11.3	0.66–0.82	0.72–0.88	Butcher et al. [2009]*
29	*E. tereticornis*	3.2–7.7	0.67–0.76	0.76–0.82	Butcher et al. [2009]*
30	*E. rudis*	6.1–7.9	0.56–0.71	0.67–0.83	Butcher et al. [2009]*
31	*E. grandis*	7–15	0.55–0.94	0.75–0.91	Faria et al. [2010]
32	*E. globulus*	5–12	0.4–0.92	0.48–0.93	Faria et al. [2010]
33	*E. urophylla*	4–10	0.13–0.86	0.48–0.92	Faria et al. [2010]
34	*E. camaldulensis*	4–14	0–1.0	0.60–0.92	Faria et al. [2010]
35	*E. dunnii*	5–9	0.5–1.0	0.74–0.91	Faria et al. [2010]
36	*E. saligna*	4–15	0.31–1	0.58–0.95	Faria et al. [2010]
37	*E. grandis*	4–7	0.25–0.81	0.05–0.87	Faria et al. [2010]
38	*E. globulus*	4–8	0.4v0.92	0.60–0.83	Faria et al. [2010]
39	*E. urophylla*	4–8	0.15–0.92	0.59–0.83	Faria et al. [2010]
40	*E. camaldulensis*	2–8	0.19–0.8	0.18–0.82	Faria et al. [2010]
41	*E. dunnii*	3–7	0.13–0.94	0.30–0.85	Faria et al. [2010]
42	*E. saligna*	4–8	0.06–0.93	0.50–0.83	Faria et al. [2010]
43	*E. aggregata*	4.66–9.33	0.65–0.71	0.70–0.73	Field et al. [2010]
44	*E. rubida*	6.67–12.8	0.75–0.79	0.736–0.91	Field et al. [2010]
45	*E. grandis*	2–7	0.06–1.0	0.18–0.77	Faria et al. [2011]
46	*E. urophylla*	1–7	0.0–0.93	0.0–0.81	Faria et al. [2011]
47	*E. globulus*	2–8	0.14–0.94	0.23–0.82	Faria et al. [2011]
48	*E. camaldulensis*	2–10	0.07–0.93	0.07–0.90	Faria et al. [2011]
49	*E. aggregata × E. rubida*	5.67–14.2	0.77–0.86	0.83–0.89	Field et al. [2010]
50	*E. pilularis*	12.6	0.78	0.75	Shepherd et al. [2010]*
51	*E. globulus*	6–21	0.71–0.93	0.5–0.9	Ribeiro et al. [2011]
52	*E. obliqua*	11–32	0.71–0.91	0.75–0.94	Bloomfield et al. [2011]
53	*E. incrassata*	-	0.61–0.92	0.76–0.80	Breed et al. [2012]
54	*E. gomphocephala*	27–60	0.58–0.73	0.6–0.69	Bradbury and Krauss [2013]
55	*E. gomphocephala*	8–27	0.52–0.87	0.51–0.84	Bradbury et al. [2013b]
56	*E. gomphocephala*	2–12	0.17–0.87	0.24–0.86	Bradbury et al. [2013a]
57	*E. victrix*	5–25	0.52–0.91	0.48–0.93	Nevill et al. [2013]
58	*E. grandis*	1–14	0–1	0.08–0.96	Zhou et al. [2014]

* Indicates the mean values; Na- Number of alleles; Ho- Observed Heterozygosity; He- Expected Heterozygosity.

### Applications of microsatellite markers in eucalypts

The stupendous and multifaceted applications of microsatellites in tree genome analysis are shown in the Figure 1. The ubiquitous nature of microsatellites in tree genome (present in nuclear, EST, mitochondria and chloroplast genome sequences) make them the most suitable DNA markers for analysis of population genetics, phylogeny and species evolution, studies on conservation and ecology and marker assisted selection/breeding (Porth and El-Kassaby [2014]). Highly informative SSR markers generate multiple alleles, thus favoring germplasm/clonal identification, genetic integrity determination during propagation and controlled breeding, seed orchard diversity, mating system and outcrossing rate analysis (Falahati-Anbaran et al. [2007]). The multilocus nature and even distribution of microsatellites over the plant genome benefit the establishment of taxonomic identity of species and phylogeny reconstruction (Ochieng et al. [2007]). Microsatellites are ideal for conservation genetics and genetic resource management because of their selectively neutral characteristics (van Zonneveld et al. [2014]) and evolutionary processes of interest to conservation geneticists (Wang et al. [2009]).

### Characterization of germplasm and individual identification

Microsatellites information on genotype is essential for effective utilization of germplasm accessions for genetic improvement for pulp yield, adventitious rooting, frost and salt tolerance, resistance to pests and diseases, efforts have continuously been made to characterize and evaluate germplasm accessions. Large amount of such germplasm and clonal accessions are maintained in tree breeding programs and clonal deployment activities of eucalypts*.* The high degree of polymorphism and the clear and simple co-dominant Mendelian inheritance of the SSRs has proven to be an extremely powerful system for the unique identification of *Eucalyptus* individuals for fingerprinting purposes and parentage testing (Kirst et al. [2005b]). Accordingly, SSR markers have been widely used for characterization of germplasm resources including clone fingerprinting, hybrid validation in controlled crosses, inter-individual genetic distance estimation, species distinction, and assignment of hybrid individuals to their most likely parent species (Stokoe et al. [2001]; Smith et al. [2003]; Grattapaglia et al. [2004]a; Kirst et al. [2005b]; Ochieng et al. [2007]; Grosser et al. [2008]; Jones et al. [2008]; Payn et al. [2008]; Rao et al. [2008]; Sampson and Byrne [2008]; Butcher et al. [2009]; Barbour et al. [2010]; Faria et al. [2010]; Shepherd et al. [2010]; Ribeiro et al. [2011]; Arumugasundaram et al. [2011]; Subashini et al. [2013]; Wheeler et al. [2013]) Clonal fingerprinting generally requires larger allele size differences for multiplexing, precise and stable allele sizing for deployment across laboratories. Hence, SSR markers with high motif length provided possibilities for greater utilization towards individual identification (Faria et al. [2011]). SSR markers play an important role in designing breeding populations and function as decision support tool in genetic conservation programs. Use of SSR markers in germplasm characterization of *Jatropha curcus* showed that the germplasm has limited diversity and the necessity of additional collections for species improvement (Alves et al. [2011]). Similarly, SSRs are the most sought after DNA markers for germplasm characterization in several tree species like avocado (Gross-German and Viruel [2013]), *Psidium guajava* (Sitther et al. [2014]), Olive (Trujillo et al. [2014]), *Tamarix* (Terzoli et al. [2014]) and *Prunus* (Khadivi-Khub et al. [2014]).

### Parentage testing and gene flow studies

Seed orchards of eucalypt species are established with individuals having high genetic diversity and low levels of inbreeding to achieve the high genetic gain in progenies. Microsatellite markers played a major role in attaining these goals by displaying mating system, degree of contamination, variation in outcrossing rates, gene flow pattern and paternal contribution in the seed orchard. The Table 6 provides the information of SSR loci used for parentage analysis and gene flow studies. Parentage assignments based on microsatellite allele information could lead to the identification of appropriate parents contributing for potential progeny (Grattapaglia et al. [2004]a). Microsatellite diversity was considered for the designing of seed orchard in *E. dunnii* (Zelener et al. [2005]) and *E. globulus* (Dini et al. [2011]) thus reducing the risks of inbreeding. *Eucalyptus* has predominantly outcrossing breeding system, nevertheless the rate of outcrossing varies among the species and populations. *E. grandis* seed orchard in exotic conditions showed the maximum outcrossing rate of 96.7% however, the pollen contamination in the seed orchard was 39.2% (Chaix et al. [2003]). Similarly, another study on *E. grandis* seed orchard in Brazil analyzed with 14 SSR markers had 8.3% selfing and 29% pollen contamination (Grattapaglia [2004]b). In native locations, *E. grandis* seed orchard populations showed a selfing rate of 14% and the paternity analysis showed that 46% seeds were due to contamination (Jones et al. [2008]). On contrary, a well isolated clonal seed orchard of *E. nitens* had an outcrossing rate of 0.85% with relatively low pollen contamination of 4.5% (Grosser et al. [2008]). A small breeding arboretum of *E. globulus* in Australia was analyzed with four highly polymorphic SSRs revealed 47.9% outcrosssing rate and 17.6% contamination rate (Rao et al. [2008]). These results indicate that microsatellites are the best tools for predicting the seed orchard performance and revealing the importance of strategies to arrest outside pollen like flowering asynchrony and isolation distance of adjacent populations. Recently, Correia et al. ([2011]) showed that tetra-, penta- and hexa nucleotide microsatellites were more informative than the di and tri nucleotide markers and specific SNPs employed for assessment of parentage and individual identification. Thus, construction of a panel of markers to assess parentage would allow considerable inputs for designing of seed orchards consequently increased productivity from half-sib eucalypt populations.

**Table 6:** **Parentage testing and gene flow studies in eucalypts**

SSR loci	Species	Parentage testing	Gene flow	Reference
Embra6, 10, 11, 13, 15, 19	*E. grandis*	✓	✓	Chaix et al. [2003]
Embra6, 10, 11, 16, 21, 22, 27, 30, 37, 40, 49,52, 53, 31	*E. grandis, E. urophylla*	✓	×	Grattapaglia et al. [2004]a
Es076, 140, 157, Eg18, 22, 26, 61, 67, 84, 86, 91, 96, 99, 126, 128, 134, En6, 16, Embra4, 6, 10, 11	*E. benthamii*	×	✓	Butcher et al. [2005]
Embra2, 4, 5, 6, 8, 10, 11, 12	*E. grandis*	✓	✓	Jones et al. [2008]
Embra5, 18, Emcrc 5, 12	E. nitens	✓	×	Grosser et al. [2008]
Emcrc5, 6, 11, Embra 10	E. globulus	✓	×	Rao et al. [2008]
FMG - EUC SSR1, 3, 5, FMRSA4, Embra3, 28, 37, 48,69, 125, 219, 227	*E. urophylla*	×	✓	Payn et al. [2008]
Embra2, 8, 10, Emcrc6, En6	*E. loxophleba*	✓	✓	Sampson and Byrne [2008]
Emcrc41, 45, 46, 47, 55, 93	*Corymbia citriodora subsp variegata*	✓	✓	Bacles et al. [2009]
Emcrc2, 7, 8, Embra10	*E. perriniana*	×	✓	Barbour et al. [2010]
Embra6, 8, 11, 12, 42, 104, 164, 187, 209, 210, 214, EPILMYB2, EPILCADP	*E. pilularis, E. pyrocarpa*	×	✓	Shepherd and Raymond [2010]
Embra914, 1284, 1382, 1445, 1468, 1990, 1928, 2002,	*E. incrassata*	×	✓	Breed et al. [2012]
EGM25, 30, 35, 47, Embra6	*E. gomphocephala*	✓	✓	Bradbury and Krauss [2013]

The natural populations of eucalypts were subjected to microsatellite based genetic analysis for gene flow estimation and population differentiation. The *E. camaldulensis* species complex in its natural range was assessed for its genetic structure with 15 microsatellite loci and concluded that the populations belongs to several subspecies with intergrade zones and breeding programs should not treat the species as a single genetic entity (Butcher et al. [2009]). Similarly, *E. globulus* species complex was surveyed with 9 microsatellite loci displayed the existence of intergrade populations and the possibilities of 4 subspecies were identified (Jones et al. [2013]). The presence of spatial genetic differentiation and large breeding zones in *E. globulus* populations in its native range revealed a distant pollen movement (Steane et al. [2006]; Yeoh et al. [2012]). Whereas, in *E. urophylla* populations of Indonesian islands, low levels of genetic differentiation across populations with high levels of gene diversity within populations were recorded (Payn et al. [2008]). Such information will have large implications in breeding of these economically important plantation species.

*Eucalyptus* species with regional distribution were analyzed for their pollen flow and pattern of genetic diversity. These studies involve microsatellite allele diversity largely indicated that in remnant and fragmented populations of *E. curtisii* (Smith et al. [2003]), *E. leucoxylon* (Ottewell et al. [2005]), *E. wandoo* (Byrne et al. [2008]), *E. benthamii* (Butcher et al. [2005]), *E. gomphocephala* (Bradbury et al. [2013a], [b]; Bradbury and Krauss [2013]), *E. incrassata* (Breed et al. [2012]) and *E. loxophleba* (Sampson and Byrne [2008]) the genetic differentiation were high across the locations and pollen based gene flow is well maintained. On the other hand, fragmentation led to high degree of clonality and inbreeding in few of the species (Smith et al. [2003]; Butcher et al. [2005]). Further, pollen being the main mode of gene flow, genetic differentiation in quantitative traits was maintained by natural selection (Bloomfield et al. [2011]). Nuclear and chloroplast microsatellite markers were deployed to examine the spatial distribution of genetic diversity in *E. pauciflora* in Tasmania and the results revealed the route of seed and pollen dispersal and population migration pattern (Gauli et al. [2014]). Effective pollen movement and spatial proximity of different species of eucalypts, which has limited reproduction barriers provides sufficient information for distances required for buffer zone in seed orchards and to maintain genetic integrity of breeding populations in exotic and natural locations. These distinctive features revealed by the microsatellite markers have a high significance in sourcing of seeds and designing breeding and conservation programs (Bacles et al. [2009]; Barbour et al. [2010]; Shepherd and Raymond [2010]).

### Linkage map and QTL identification

Microsatellite markers are regarded to be the tools in marker assisted selection and they are widely utilized for genetic mapping in many forest trees and they are extremely suitable for QTL localization and comparative mapping purposes. Detailed information on the genetic maps developed till date was reviewed by Grattapaglia et al. ([2012]). Most of the genetic mapping studies in eucalypts targeted the commercially important species such as *E. grandis*, *E. urophylla*, *E. grandis*, *E. globulus*, *E. camaldulensis* and *E. nitens* (Bundock et al. [2000]). The Additional file 1: Table S1 shows the SSR markers mapped in genetic linkage maps of different species. In eucalypt inter-specific hybrids, the first genetic map was developed with dominant RAPD markers using pseudotestcross approach (Grattapaglia and Sederoff [1994]). Immediately after the development of genetic maps, QTL localization for growth, adventitious rooting and wood properties were carried out rapidly. Microsatellite linkage mapping was possible only in 1998 for the *Eucalyptus grandis* × *E. urophylla* interspecific cross with 20 highly informative Embra SSR loci (Brondani et al. [1998]). Congruity of genetic linkage maps of different eucalypt species had an advantage of consolidating linkage groups across species and quantitative trait loci influencing the traits of interest. The marker correspondence across linkage grouping and position of the SSR loci across genetic maps of eucalypt species was almost similar with few exceptions (See Additional file 1 for linkage group information). The first QTL study involving SSR loci in *Eucalyptus* for a series of wood properties was reported by Thamarus et al. ([2002]). Vegetative propagation traits were located on homeologous linkage groups of *E. grandis*, *E. urophylla*, *E. tereticornis* and *E. globulus* (Marques et al. [2002]). Further, integrated linkage maps with different types of DNA markers were developed for many species of eucalypts. The pure species genetic map for *E. camaldulensis* was developed with RAPDs, RFLPs and SSRs by selecting highly divergent parent trees for mapping population generation (Agrama et al. [2002]). With the development of new SSR markers, Brondani et al. ([2006]) could generate a comprehensive consensus linkage map by including SSR loci information from various eucalypts species. In the recent years, along with the next generation markers like SNPs, SFPs and DArT markers, SSRs are used as framework markers to confirm the linkage groups and position of markers. The SSR markers were used to estimate linkage disequilibrium in eucalypts but reported to be lasting very fast with every 200 bp approximately (Arumugasundaram et al. [2011]). However, in several instances SSR loci were found to be in close association with quantitative traits. For example, in *E. grandis* breeding population Embra125 and Embra1071 were found to be in linkage equilibrium with rust resistance loci *Ppr* 1 at 9.5 and 7 recombination, respectively (Mamani et al. [2010]). Embra125 loci was found to be closely linked with rust resistance in *E. grandis* [(*E. grandis*) × (*E. urophylla* × *E. grandis*)] explaining 42% of the phenotypic variation (Rosado et al. [2010]). Recently, between eSSR markers Embra1656 and Embra1071 (16.4 and 1.4 cM away, respectively) the QTLs governing moderate proportion of the genetic variation (11.5%) for rust resistance was identified (Alves et al. [2011]).

In *E. globulus* putative QTL for *Mycosphaerella cryptica* resistance was closely associated with microsatellite marker Embra48 (Freeman et al. [2008b]). Similarly, Embra12 was in closely linked to foliar concentrations of terpenes and formylated phloroglucinol compounds in *E. nitens* and *E. globulus* (Henery et al. [2007]; Freeman et al. [2008a]). Embra173 alone explained 53.8% variation for formylated phloroglucinols in the foliage of *E. globulus* (Freeman et al. [2008b]). In another study by Freeman et al. ([2009]) in *E. globulus* several Embra SSR loci were linked to wood properties and growth traits. Additive and dominant QTLs were found in tight linkage with SSR markers tested for drought tolerance in *E. grandis* × *E. urophylla* hybrid clones (Teixeira et al. [2011]).

The presence of generic genomic regions was validated through SSR markers, which enabled the identification of orthologous QTL regions for wood properties in *E. nitens* and *E. globulus* (Thumma et al. [2010]) and *E. urophylla* and *E. grandis* (Gion et al. [2011]). Genic SSRs were found to be largely associated with economically important traits in many plant species. *Eucalyptus* transcriptome sequencing projects led to the development of many eSSR loci and many were used in genetic maps (Faria et al. [2011]). *Eucalyptus* species have valuable SSR resources for comparative genomic studies and they also serve as framework markers for construction of a consensus map across species. Addition of fully informative microsatellites on the framework map permits linkage map homology, QTL and candidate gene positions across different eucalypt species (Freeman et al. [2009]). *Eucalyptus* SSRs were proved again for their colinearity across different species and linkage to physical correspondence on the reference genome sequence (Grattapaglia et al. [2014]).

### Future prospects

Microsatellites have major roles to play in various spheres of eucalypts genetics and improvement. They are one of the three major classes of genetic variations along with SNPs (single nucleotide polymorphisms) and CNVs (copy number variations) and have many important biological functions (Gemayel et al. [2010]). Recent evidences suggest that variations in microsatellites may lead to phenotypic changes (Joy and Soniya [2012]) and adaptive evolution (Fidalgo et al. [2006]). The available genome sequence of the eucalypts genome does not diminish the importance of microsatellites, as these markers will extend annotated genome resources of sequenced *Eucalyptus* species to genetic study/breeding in different eucalypt species.

Genomic SSRs have a broad range of applications, and in particular being neutral markers, which are not linked to any particular trait, but most probably offer a representation of the underlying genetic diversity in wild populations and to target populations for conservation (van Zonneveld et al. [2012]). Further, microsatellites enable the rapid identification of cryptic species and have been used successfully to identify species hybrids in many tree species including eucalypts. They contribute to a better understanding of the processes involved in the development of contemporary patterns of variation, including the regional contraction and expansion of populations and refugia (Nevill et al. [2014]).

Several reports confirmed the presence of SSRs in transcription factors and promoters of genes for facilitating transcriptional plasticity. Hence, identification of new gSSRs and eSSRs would pave way for better understanding of the *Eucalyptus* genome. Genic SSR markers could represent the new class of functional markers, finding use in evolutionary studies, comparative mapping, candidate gene association mapping, gene discovery and molecular breeding (Shi et al. [2014]). In silico SSR polymorphism analysis, a novel access to selecting polymorphic markers is currently advocated to reduce the cost and to increase the efficiency of SSR development.

Further, enough care should be exercised while handling null alleles, imperfect repeats, and allelic dropout, equally they can lead to an overestimation of observable alleles, a decrease in observed heterozygosity, and an increase in the apparent level of inbreeding. On the basis of microsatellite analysis, spatial genetic structure (SGS) can be estimated to delineate provenances of eucalypts. In addition, non-denaturing FISH (ND-FISH) can be used to compare the distribution of SSRs to determine whether the range of molecular diversity shown by these highly polymorphic sequences is reflected at the chromosome level. They are also ideally used as anchor markers in molecular linkage maps and in generation of consensus maps across species and that can be highly saturated with DArT and SNP/genotyping by sequencing markers (GBS). Development of a high-density consensus genetic map with SSRs in an important chromosomal interval provides eucalypt molecular breeding programs with a better choice regarding the quality of markers and a higher probability of polymorphic markers. They play a major role in aligning linkage map due to their high transferability and have a functional role in trait variation and to see the conservation and diversification of gene order across the species of eucalypts.

## Conclusions

Microsatellite markers play a major role in eucalypts at different levels of genetic improvement. The inherent potentiality of these marks to distinguish closely related individuals is increasingly encouraging for the mining of more and more SSRs for placing on linkage groups and other genetic studies. Microsatellites containing DNA sequences and their functional role in the eucalypt genome were investigated and detected linkage-to-physical position for a large number of microsatellites. Recently, two hundred and twenty three new microsatellite markers were surveyed for allelic diversity and added to the existing eucalypt SSR map, bringing the total number of genetically mapped SSR loci to >400 and strengthening the comparative genome mapping (Grattapaglia et al. [2014]).

Additionally, the miRNA-SSR markers, i.e., presence of SSRs in precursors of miRNA candidates (Joy et al. [2013]) brought a new biological significance to microsatellites, wherein the microRNAs (miRNAs) play a major role in post transcriptional gene silencing. The length variation of the SSRs in salt responsive miRNA genes provided sensitivity to salinity adaptation of *Oryza sativa* (Mondal and Ganie [2014]). Any prosperous utilization in tree breeding, SSRs is required in greater numbers. Therefore, recognition of such miRNA-SSRs in eucalypts would lead to better understanding of their role in post-transcriptional gene regulations and phenotypic variations.

## Additional file

## Electronic supplementary material

Additional file 1: Table S1.: Linkage group information for SSR loci in eucalypts. (XLS 546 KB)

Below are the links to the authors’ original submitted files for images.

Authors’ original file for figure 1

Authors’ original file for figure 2
