# Supplementary figures and images for: Microsatellite resources of Eucalyptus: current status and future perspectives
Source: Bot Stud. 2014 Oct 25;55:73. doi: 10.1186/s40529-014-0073-3 (PMC5430318; doi:10.1186/s40529-014-0073-3)

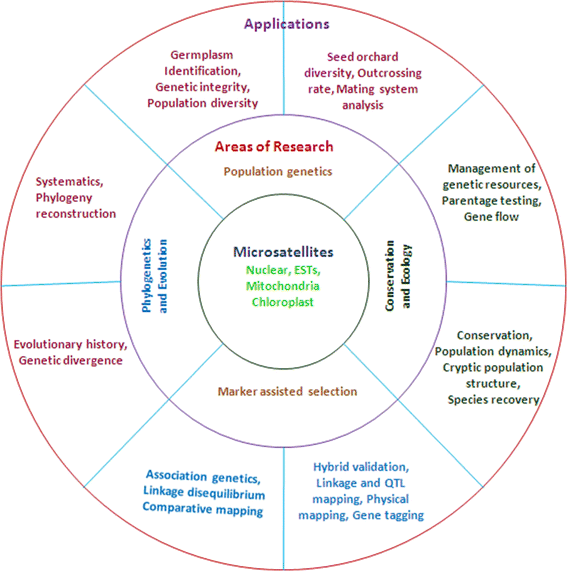

Supplement: Supplementary file 2 — Authors’ original file for figure 1 [file 40529_2014_9073_MOESM2_ESM.gif]

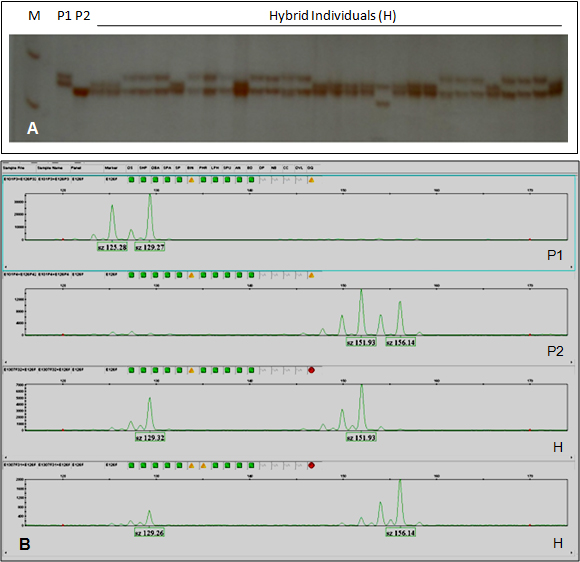

Supplement: Supplementary file 3 — Authors’ original file for figure 2 [file 40529_2014_9073_MOESM3_ESM.jpeg]
